# Partitioning of net CO_2_ exchanges at the city-atmosphere interface into biotic and abiotic components

**DOI:** 10.1016/j.mex.2021.101231

**Published:** 2021-01-13

**Authors:** Keunmin Lee, Je-Woo Hong, Jeongwon Kim, Jinkyu Hong

**Affiliations:** a Ecosystem-Atmosphere Process Laboratory, Department of Atmospheric Sciences, Yonsei University, Seoul, South Korea; b Korea Environment Institute, Sejong, South Korea

**Keywords:** Building emission, Carbon cycle, CO_2_ flux, Ecosystem respiration, Eddy covariance, Partitioning, Photosynthesis, Urban forest, Vehicle emission

## Abstract

Eddy covariance (EC) method has been used to measure CO_2_ fluxes over various ecosystems. Recently, the EC method has been also deployed in urban areas to measure CO_2_ fluxes. Urban carbon cycle is complex because of the additional anthropogenic processes unlike natural ecosystems but the EC method only measures the net sum of all CO_2_ sources and sink. This limitation of the EC method hinders us from the underlying processes of the carbon cycle, and it is necessary to partition the net CO_2_ fluxes into individual contributions for a better understanding of the urban carbon cycle. Here we propose a statistical method to partition CO_2_ fluxes into individual components by extending the method of Menzer and McFadden (2017).

• Statistical method is proposed to partition CO_2_ fluxes into gross primary production, ecosystem respiration, anthropogenic emissions from a vehicle and building.
• This method uses eddy fluxes and footprint-weighted high-resolution land cover data with temporal subsets that a few components can be negligible.
• New partitioning method produces reliable individual components of the urban carbon cycle when compared to inventory data and typical biotic responses to environmental conditions.

## Graphical abstract

**Figure:**
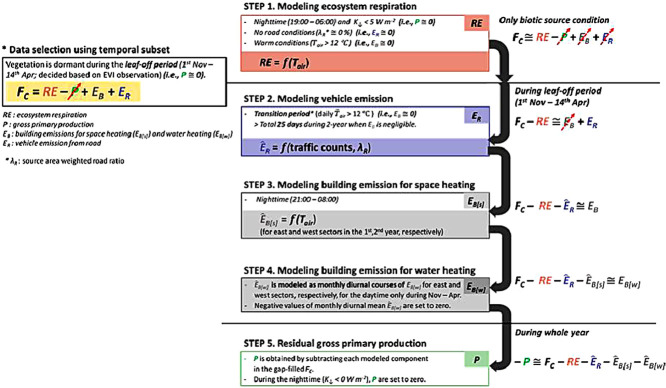

Specifications table

**Table:** 

Subject Area	Earth and Planetary Sciences
More specific subject area	Urban Climate, Microclimate, Flux measurement, Carbon Cycle
Method name	CO_2_ flux partitioning in urban area
Name and reference of original method	Crawford and Christen [1]. Spatial source attribution of measured urban eddy covariance CO_2_ fluxes. Theoretical and applied climatology, 119(3–4), 733–755.
	Menzer and McFadden [12]. Statistical partitioning of a three-year time series of direct urban net CO2 flux measurements into biogenic and anthropogenic components. Atmospheric Environment, 170, 319–333.
Resource availability	

## *Method details

Urban CO_2_ budget is formulated as:

(1) $$F_{C}=RE-P+E_{R}+E_{B}$$

where *F_C_* is the net CO_2_ exchange at the city-atmosphere interface, *RE* is the ecosystem respiration, *P* is the gross primary production by vegetation, and *E_R_* and *E_B_* are the anthropogenic CO_2_ emissions from fossil fuel combustions by vehicles and buildings, respectively. It is important to partition *F_C_* into individual components (i.e., *RE, P, E_R_*, and *E_B_*) to investigate the controlling factors of the urban carbon cycle in a changing environment. In natural canopies and croplands, *RE* is estimated by modeling the relationship between the EC-measured nighttime *F_C_* and air or soil temperature, and then *P* is estimated by subtracting this modeled *RE* from *F_C_* (e.g., [15]).

**Fig. 1:**
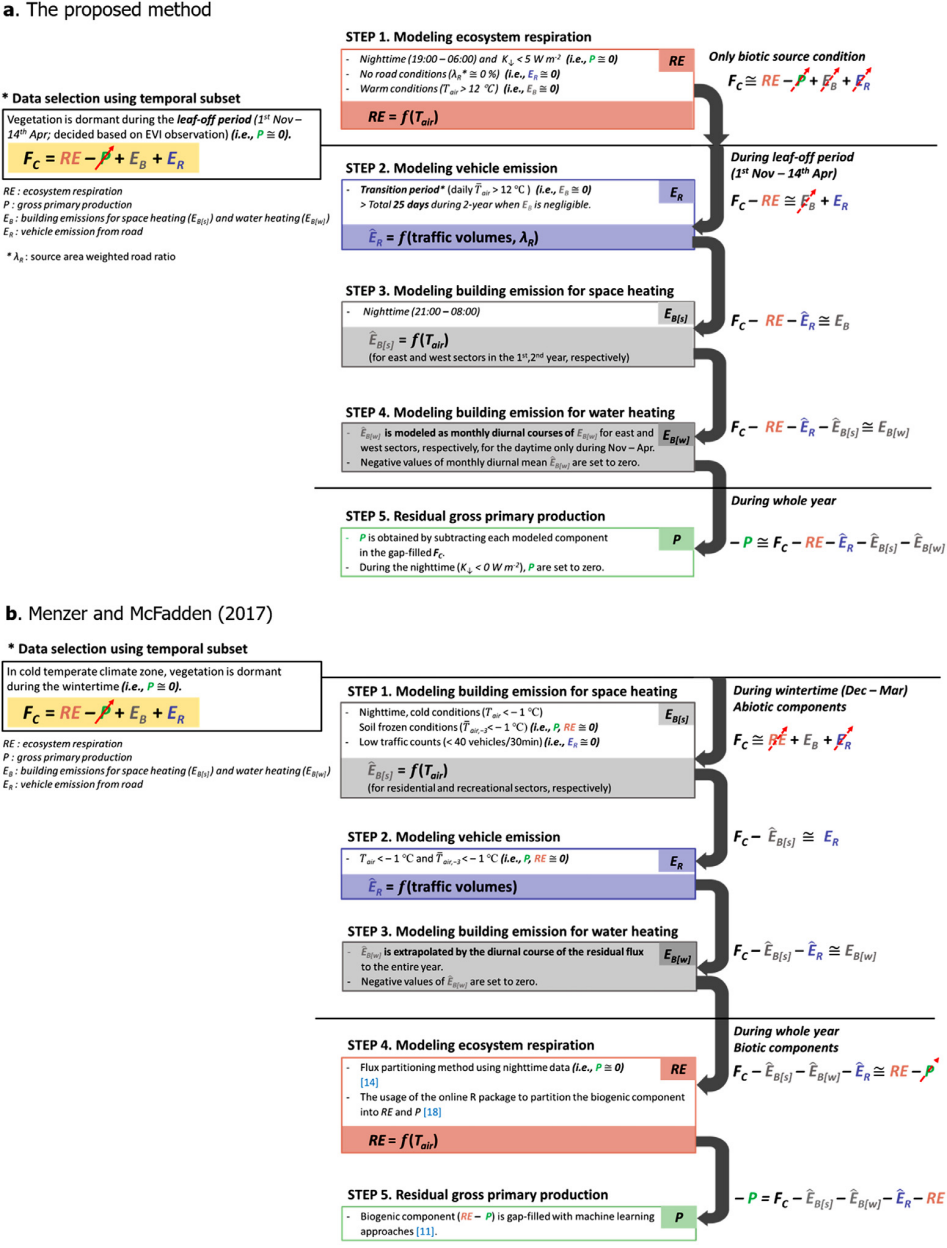
Schematic diagram of (a) the proposed method in this study and (b) Menzer and McFadden [11], [12], [14], [18] to partition CO_2_ flux (*F_C_*) into four components: ecosystem respiration (*RE*), gross primary production (*P*), and anthropogenic CO_2_ emissions by vehicles (*E_R_*) and buildings (*E_B_*). The hatted variables are the modeled components in Eq. (1). The data filter conditions used in the process of modeling each component are not universal, and it is recommended to set the criteria according to the characteristics of each observation site.

However, unlike natural ecosystems, there are extra anthropogenic CO_2_ sources (i.e., *E_R_* and *E_B_*) at the city-atmosphere interface. Thus, additional information and process are required for the *F_C_* partitioning into each CO_2_ sources and sink. For example, Pataki et al. [13] used stable isotope measurement data for the partitioning. Meanwhile, Crawford and Christen [1] and Menzer and McFadden [12] proposed statistical methods for the *F_C_* partitioning in urban areas. Crawford and Christen [1] partitioned *F_C_* by using meteorological variables and the flux footprint weighted land cover climatology in suburban area where vehicle emission is a dominant source. Menzer and McFadden [12] partitioned *F_C_* by modeling the anthropogenic components with traffic volume and air temperature (*T_air_*) based on winter *F_C_* data when only anthropogenic sources contribute to net CO_2_ fluxes. Our new proposition extends the statistical method of Menzer and McFadden [12] (MM2017 hereafter) by combining MM2017 with the flux footprint weighted land cover climatology based on Crawford and Christen [1] for the urban CO_2_ flux partitioning. Similarly to MM2017, our method selects specific temporal subsets in that some components in Eq. (1) are negligible. Additionally, our method takes advantage of traffic volume data and the footprint weighted road cover fraction (*λ_R_*). Accordingly, our proposed method is applicable to more general urban conditions especially when *E_R_* is the main source from the heavy traffic volume in a city. Fig. 1 shows the schematic flow chart to partition net CO_2_ exchange into contributing components by our method with the MM2017 for comparison. Here we explain the procedures of our method with an example EC data observed in an artificially constructed urban forest [4,^8^,9].

## Data preparation

Our partitioning method requires the EC data with other ancillaries of the footprint-weighted land cover fraction (λ), traffic volumes, and vegetation index (e.g., EVI: Enhanced Vegetation Index). λ is estimated by overlaying flux footprints on the high-resolution land cover map, which needs the atmospheric stability estimate from the EC data, a footprint model, aerodynamic roughness parameters, and the morphological data of digital surface and terrain models.

In the example case, the 30-min CO_2_ flux is computed using the EddyPro software (version 6.2.0, LI-COR, USA) from 10 Hz sampled raw data and the post data process for quality control ([3] and references therein). Footprint climatology is computed using the footprint model of Hsieh et al. [2] and a 1 m-resolution land cover map provided by Environmental Geographic Information Service (https://egis.me.go.kr) following Kim et al. [6]. In the land cover map, the land cover was classified into tree, grass, building, road, impervious, water, bare soil, and the other. As inputs for the footprint model, aerodynamic roughness parameters such as roughness length and zero-plane displacement height are estimated using the morphological data of digital surface and terrain models provided by National Geographic Information Institute (https://www.ngii.go.kr) [4,8] at 1 × 1 m^2^ resolution. Eventually, λ is computed by the summation of each footprint-weighted land cover [1]. To estimate *E_R_* over the heterogeneous urban area, *E_R_* is modeled with traffic volumes and footprint-weighted road cover fraction (*λ_R_*) in our method. This procedure presumes that *E_R_* is proportional to its corresponding surface type fraction (i.e., *λ_R_*). In the example, the hourly traffic volume is counted on a road near the flux tower (https://topis.seoul.go.kr).

## Partitioning procedures

In statistical partitioning methods, it is important to select relevant temporal subsets of *F_C_* data so that components in Eq. (1) are negligible. For example, MM2017 chose wintertime *F_C_* with low traffic volume in cold temperate climate zone so that *P* and *RE* were negligible and *E_R_* was also small, thus only *E_B_* contributed to *F_C_* (Step 1 in Fig. 1a). However, in a typical metropolis, such condition is not always feasible because it is probable that traffic volume is high in the footprint areas. Our method is designed to estimate *E_R_* in such complex conditions (Table 1). The following sections describe the procedure to partition *RE, E_R_, E_B_*, and *P* in detail.

**Table 1:** Comparison between method of (a) the proposed method in this study and (b) Menzer and McFadden [12]. The parameters used to model each component are values that are only applicable to their corresponding site.

Partitioning method	(a) The proposed method	(b) Menzer and McFadden [12]
Partitioning order	*RE* → *E_R_* → *E_B(s)_* → *E_B(w)_* → *P*	*E_B(s)_* → *E_R_* → *E_B(w)_* → *RE* → *P*
Input variables	*T_air_, Traffic volumes, λ_R_*	*T*_*air*_*, Traffic volumes*
Used temporal subset	Transition period Leaf-off period	Wintertime
Partitioned component	*E*_*R*_*E*_*B(s)*_*, E*_*B(w)*_	*E*_*B(s)*_*, E*_*R*_*, E*_*B(w)*_
Example site	Seoul Forest Park in Seoul, Korea (*37°32’41’’N, 127°2’16’’E*)	KUOM in Minnesota, USA (*44°59’N, 93°11’W*)
Vegetation cover / Local climate zone (LCZ, [16])	46.6% / LCZ_A_	82% / LCZ_6_
Traffic volume [vehicles d^−1^]	~ 100,000	~ 10,000
Parameters for *E_R_* [μmol m^−2^ s^−1^ 30-min veh^−1^]	0.0798 × *traffic volumes* × *λ_R_* (unstable)0.0666 × *traffic volumes* × *λ_R_* (neutral)0.0319 × *traffic volumes* × *λ_R_* (stable)	0.0053 × *traffic volumes*
Parameters for *E_B(s)_*	−0.293 × *T_air_* + 3.638 (west/2nd year)−0.128 × *T_air_* + 0.413 (east/1st year)−0.198 × *T_air_* + 1.344 (east/2nd year)−0.293 × *T_air_* + 3.638 (west/2nd year)−0.128 × *T_air_* + 0.413 (east/1st year)	−0.201 × *T_air_* + 4.054 (residential)−0.136 × *T_air_* + 3.108 (recreational)−0.201 × *T_air_* + 4.054 (residential)

### Estimation of ecosystem respiration

First, *RE* is computed by the van't Hoff-type regression of nighttime *F_C_* to *T_air_* [17] because *RE* has the exponential relationship with *T_air_* generally. This nonlinear regression should be limited to the periods of negligible *P, E_R_*, and *E_B_*: 1) *P* is zero during nighttime, 2) *E_R_* is negligible if the source area weighted road fraction is small (i.e., *λ_R_* ≅ 0), and 3) *E_B_* is insignificant in the warm season because people stop the use of gas-based heating systems.

**Fig. 2:**
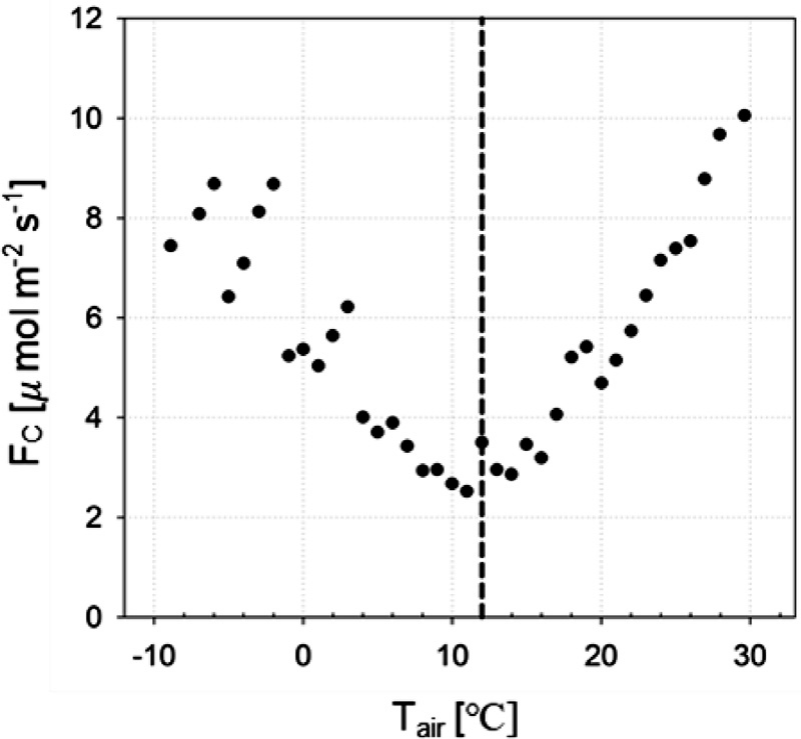
The relationship between nighttime net CO*_2_* exchange (*F_C_*) in the urban forest and air temperature (*T_air_*) in bins of every 1 °C. Air temperature of 12.0 °C at which building emissions (*E_B_*) are negligible is determined by the changing point analysis [5].

**Fig. 3:**
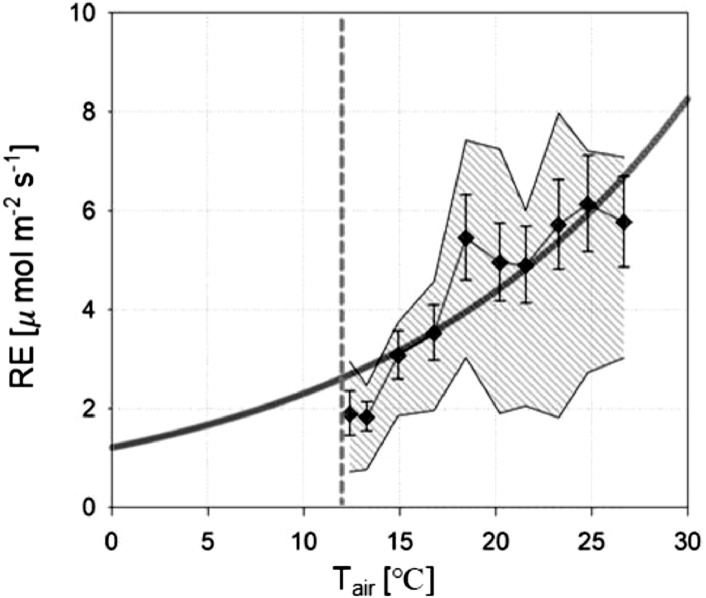
The temperature response curve of the nocturnal  CO*_2_* flux (*F_C_*) having negligible gross primary production (*P*), vehicle emissions (*E_R_*), and building emissions (*E_B_*). Each bin is 40 samples. The error bars and the shaded area represent standard errors and interquartile range, respectively.

In the example case, nighttime data is selected if downward shortwave radiation (*K_↓_*) is below 5 W m^−2^ during the sunset periods, and the warm season is defined as the period of *T_air_* > 12.0 °C which is determined by the changing point analysis from the exponential decreases to increases of *F_C_* to *T_air_* based on Killick et al. [5] (Fig. 2). The final estimation of *RE* is as follows (Fig. 3):

(2) $$RE=1.236\times e^{\left(0.063\;\;\times \;\;T_{\text{air}}\right)}\left(\text{unit}:\;\mu \text{mol}\;m^{-2}s^{-1};\;r^{2}=0.78\right)$$

*Q*_10_ (the rate by which respiration is multiplied when temperature increases by 10 °C) is approximately 1.9 at the example site and matched the *Q*_10_ value for ecosystem respiration (2.2 ± 0.7) calculated in natural forests across 42 FLUXNET sites [10].

### Estimation of anthropogenic CO_2_ emissions from fossil fuel combustions by vehicles

*E_R_* is estimated by the data during the transition period (hereafter TP) defined by the period of both negligible *E_B_* and *P*. TP corresponds to the leaf-off period with the relatively warm temperature and no space heating. That is, during TP, *F_C_* consists of only *RE* and *E_R_* and so *E_R_* is simply obtained by subtracting the modeled *RE* (i.e., Eq. (2)) from *F_C_*.

**Fig. 4:**
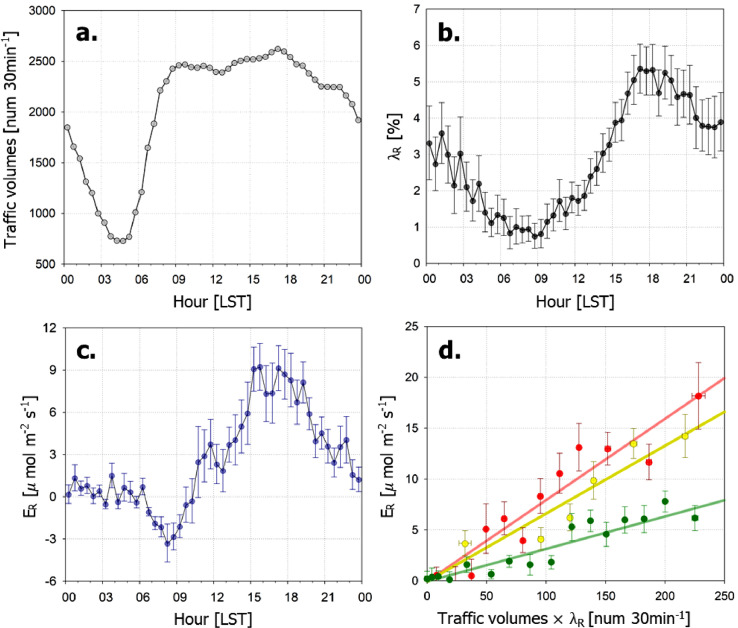
Mean diurnal variations of (a) traffic volume, (b) the source area weighted road ratio (*λ_R_*), and (c) vehicle emissions (*E_R_* ≅ *F_C_* – *RE*) during the transition period. (d) The relationship between *E_R_* and traffic volumes multiplied by *λ_R_*. The lines in (d) are modeled *E_R_* in unstable condition (*z_u_*/*L* ≤ – 0.04; red), neutral condition (|*z_u_*/*L*| < 0.04; yellow), and stable condition (*z_u_*/*L* ≥ 0.04; green), respectively (*z_u_* = *z*’ × [log(*z*/*z*_0_) – 1 + *z*_0_/*z*], *z*’ = *z_m_* – *z_d_, z*_0_: roughness length, *z_m_*: measurement height, *z_d_*: zero-plane displacement height, and *L*: Obukhov length). Points in (d) are averages of 20 samples in ascending order from the highest traffic volumes × *λ_R_*. The error bars represent standard errors.

In the example case, the leaf-off period (1st November*–*14th April) is decided based on EVI, and TP is selected as total 25 days for two years with daily mean *T_air_* > 12.0 °C in this leaf-off period. In previous studies, only traffic volume was used as an input variable to predict *E_R_*, but *E_R_* shows diurnal variation concurrently with traffic volume and *λ_R_* (Fig. 4a–c). Indeed, *E_R_* shows the best correlation with traffic volume multiplied by *λ_R_*. We binned the data by averaging every 20 samples in ascending order from the lowest traffic volumes multiplied by *λ_R_* and regressed them against *E_R_* with the atmospheric stability (Fig. 4d):

(3) $${\hat{E}}_{R}=0.0798\times \text{traffic}\;\text{volumes}\;\times \;\lambda_{R}\left(\text{unstable};\;r^{2}=\;0.88\right)$$

(4) $$\;=0.0666\times \text{traffic}\;\text{volumes}\;\times \;\lambda_{R}\left(\text{neutral};r^{2}=0.86\right)$$

(5) $$\;=0.0319\times \text{traffic}\;\text{volumes}\;\times \;\lambda_{R}\left(\text{stable};r^{2}=0.89\right)$$

In Eqs. (3)–(5), the slope coefficients correspond to a CO_2_ emission rate per vehicle per half-hour (μmol m^−2^ s^−1^ 30-min veh^−1^). The estimated slopes are comparable to the inventory data. That is, the average monthly traffic speed for the road is 50–60 km h^−1^ (Seoul Metropolitan Government Traffic Speed Report), and CO_2_ emission rate is approximately 150 g CO_2_ km^−1^ veh^−1^ based on the emission data [7]. With the width of the eight to ten-lane road (25–30 m), these estimated slopes are in the range of 0.0631–0.0758 μmol m^−2^ s^−1^ 30-min veh^−1^ accordingly (≅ 150 gCO_2_ km^−1^ veh^−1^ × 1/30 or 1/25 m^−1^ × 1/44 mol gCO_2_^−1^ × 10^−3^ km m^−1^ × 10^6^ μmol mol^−1^ × 1/1800 30-min s^−1^).

### Estimation of anthropogenic CO_2_ emissions from fossil fuel combustions in buildings

After estimation of *RE* and *E_R_, E_B_* can be estimated by subtracting them from the observed *F_C_* during the leaf-off season (i.e., *P* ≅ *0*). *E_B_* consists of emissions from space heating (*E_B(s)_*) and from water heating (*E_B(w)_*). In general, *E_B(s)_* is proportional to *T_air_* because natural gas consumption for space heating increases as it gets colder, and *E_B(w)_* is small in nighttime. Accordingly, nighttime *E_B_* data consists of *E_B(s)_* only and *E_B(s)_* is estimated from the relationship between nighttime *E_B_* and *T_air_*.

**Fig. 5:**
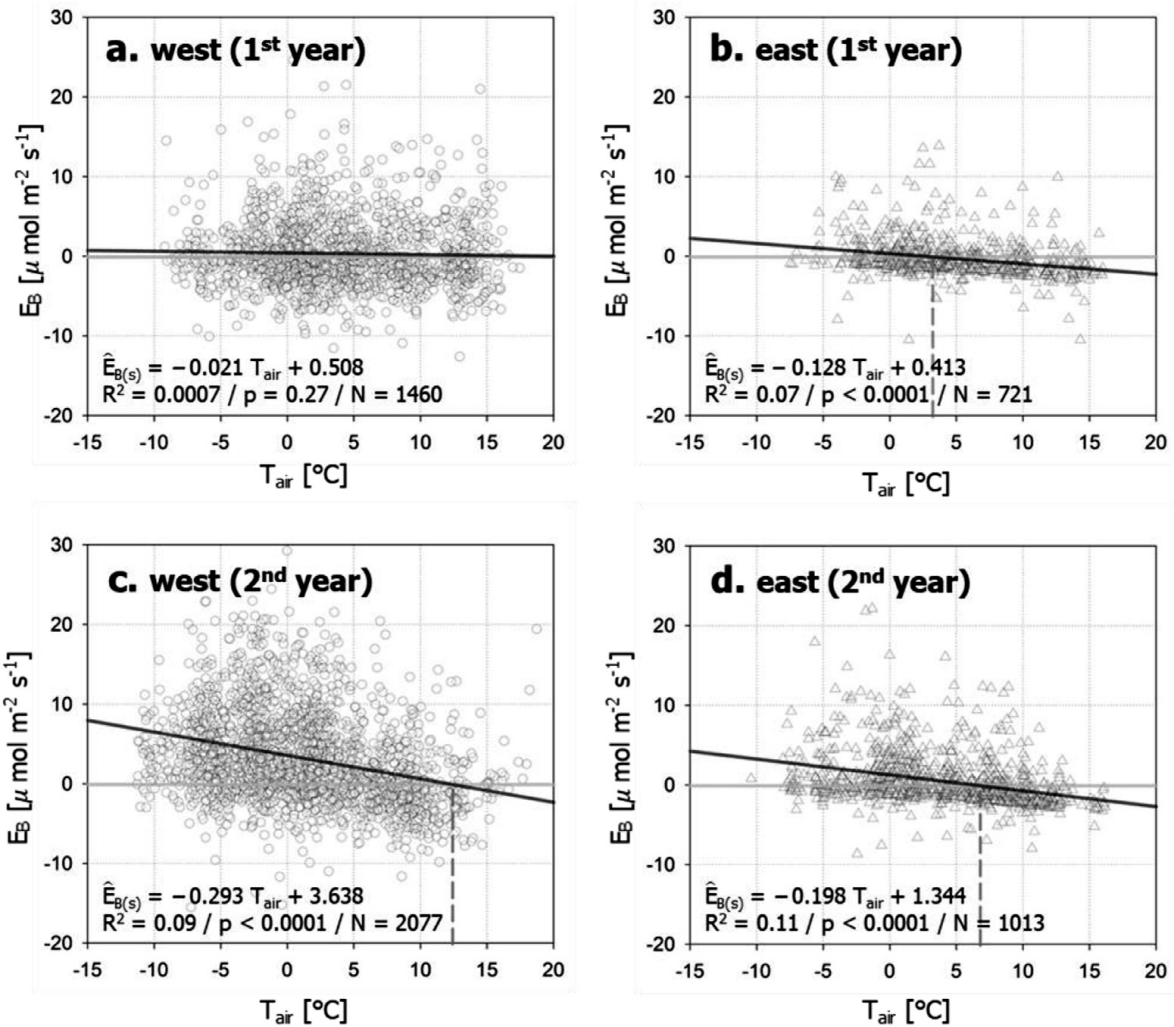
The relationships of nighttime building emissions (*E_B_*≅*F_C_* – *RE* – *E_R_*) with *T_air_* on the western side (wind direction > 120°) (a and c for the 1st and 2nd year, respectively) and on the eastern side (wind direction < 120°) (b and c for the 1st and 2nd year, respectively). The dashed lines represent *T_air_* where modeled *E_B__(s)_* is zero. The modeled *E_B__(s)_* is indicated within each panel, and *R*^2^ is the coefficient of determination of the linear regression, and the *p*-value is for the slope coefficient.

In the example case, nighttime *E_B(w)_* is also small because the urban park is closed at night (from 21:00 to 08:00), thus leading to no usages of hot water. Particularly, in the example case, *E_B(s)_* is estimated separately for the first and second year, and on the western and the eastern side, respectively to incorporate spatio-temporal variations in *E_B(s)_* around the measurement location (Fig. 5):

(6) $${\hat{E}}_{B\left(s\right)}=-\;0.021\;\times \;T_{\text{air}}\;+\;0.508\;\;\left(\text{western}\;\text{side},\;1\text{st}\;\text{year};\;p\;=\;0.29\right)$$

(7) $$\;\;=-\;0.293\times T_{\text{air}}+3.638\;\;\left(\text{western}\;\text{side},\;2\text{nd}\;\text{year};\;p<0.0001\right)$$

(8) $$\;=-\;0.128\times T_{\text{air}}+0.413\;\;\;\left(\text{eastern}\;\text{side},1\text{st}\;\text{year};\;p<0.0001\right)$$

(9) $$\;=-\;0.198\times T_{\text{air}}+1.344\;\;\;\left(\text{eastern}\;\text{side},2\text{nd}\;\text{year};\;p<0.0001\right)$$

**Fig. 6:**
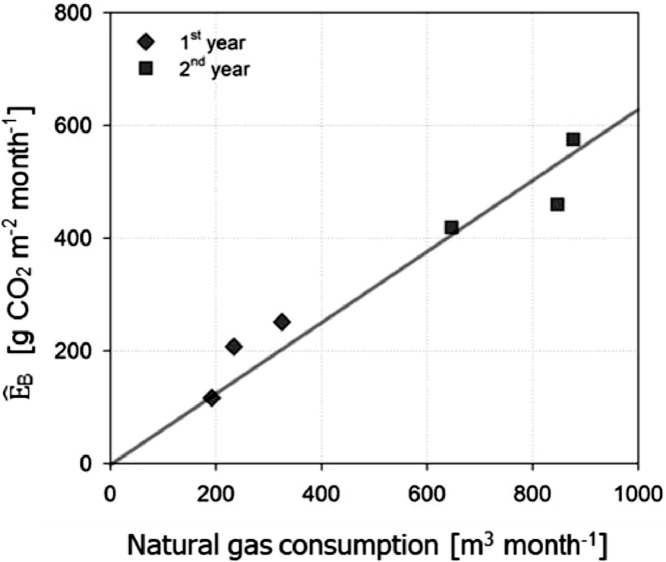
The relationship of monthly gas consumption in the park facility for heating and monthly sum of modeled building emissions ($\hat{E}$*_B_* = $\hat{E}$*_B(s)_* +$\hat{E}$*_B(w)_*) in the winter season.

The slopes in Eq. (6)–(9) are the sensitivity of *E_B(s)_* to *T_air_* and their annual changes match variations in annual gas consumption recorded at the park facility building around the tower. In the example, a direct emission source by space heating is located on the western side of the tower and is larger in the second year than the first year based on the records at the facility building around the tower. Notably, these annual changes in the slopes match the larger slope in Eq. (7) compared to that in Eq. (6) (Fig. 6).

**Fig. 7:**
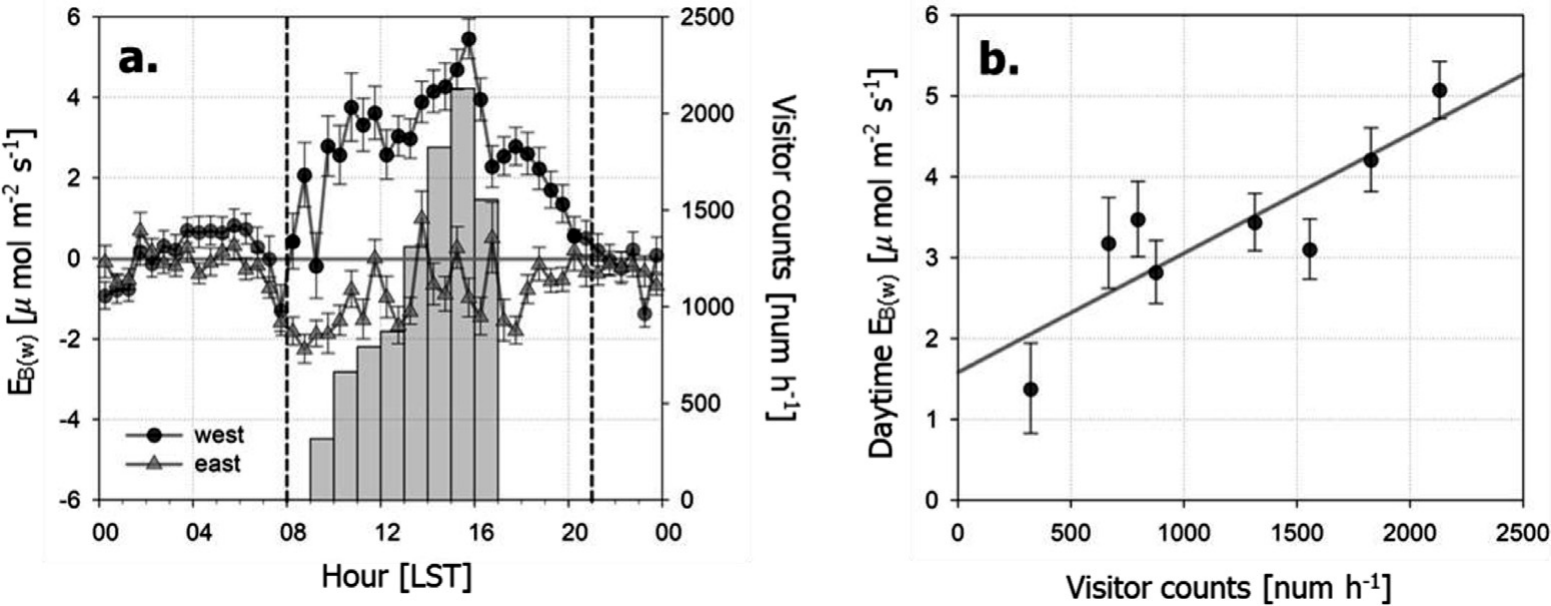
During leaf-off period, (a) mean diurnal patterns of park visitor counts (*bars*) and building emissions for water heating (*E_B(w)_*≅*F_C_* – *RE* – $\hat{E}$*_R_* – $\hat{E}$*_B(s)_*) on the western (wind direction > 120°; *black circles*) and eastern side (wind direction < 120°; *gray triangles*). (b) The relationship between park visitor counts and daytime hourly *E_B(w)_* on the western side from 09:00 to 17:00. The dashed line in (a) represents the daytime (08:00 to 21:00) when visitors use park facilities. The error bars represent standard errors.

There is still non-zero residual during the leaf-off season with its diurnal variation, which can be attributed to *E_B(s)_* and errors. In our example, on the western side, it shows a clear diurnal pattern and follows the number of park visitors but the eastern side residuals change their sign for a day (*R*^2^=0.74, Fig. 7). Our results suggest that this residual mainly comes from *E_B(s)_* in daytime but error is dominant in case of no significant CO_2_ sources (i.e., the eastern side in the example). Furthermore, the monthly sum of the modeled *E_B_* is comparable to the estimation based on the monthly gas consumption data of the park facility during the winter with a significant correlation (*R*^2^ = 0.94), indicating that our method is more reliable in monthly timescale (Fig. 6).

### Estimation of gross primary production

**Fig. 8:**
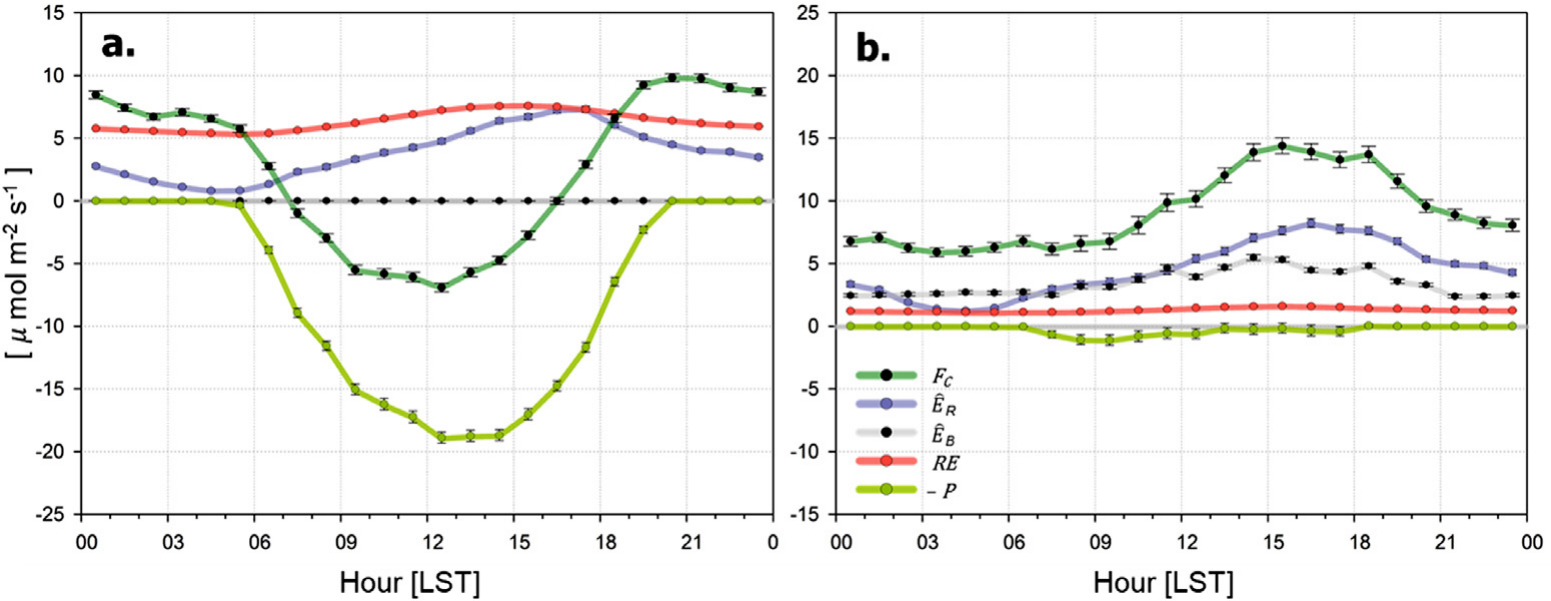
Mean diurnal variations of CO_2_ flux (*F_C_*), vehicle emissions (${\hat{E}}_{R}$), building emissions (${\hat{E}}_{B}$), ecosystem respirations (*RE*), and gross primary production (*P*) in (a) summer and (b) winter seasons.

After *RE, E_R_* and *E_B_* are modeled sequentially, *P* is obtained as the residuals by subtracting the remaining components from the observed *F_C_* (Fig. 8):

(10) $$P=\mathrm{RE}+{\hat{E}}_{R}+{\hat{E}}_{B}-F_{C}$$

*_._* In this step, it is prerequisite to fill the missing in *F_C_* and small negative *P* (<∼ 2 μmol m^−2^ s^−1^) at night is replace with zero. Further investigation reveals that 10% errors in turbulent fluxes and their related uncertainties in the flux footprint produces *P* uncertainty of < 1% only.

## Concluding remarks

This study proposes a new statistical methodology for separating the eddy covariance CO_2_ flux observed in a complex urban environment with air temperature, traffic volumes, and land cover information. Our proposed method can be used for urban interfaces influenced by heterogeneously distributed CO_2_ sources and sink where previous statistical methods are inapplicable. Our method is applied for an example data observed at an urban forest where roads are adjacent to the measurement location and provides comparable estimation to the inventory data. In typical urban environment where CO_2_ sources and sinks have spatiotemporally complex distribution, our methodology can provide reliable estimations of CO_2_ sources and sink and improve our understanding of urban carbon cycle accordingly and our estimations are insensitive to uncertainties in measurement and ancillary data. More extensive observation and validation studies are required to investigate impacts of uncertainties in the footprint model and EC data on the flux partitioning. It is also notable that our estimated empirical parameters are site-specific and need independent estimation in other urban sites.

## Declaration of Competing Interest

The authors declare that they have no known competing financial interests or personal relationships that could have appeared to influence the work reported in this paper.
